# Psychological Stress and Venous Thromboembolism: A Narrative Overview

**DOI:** 10.3390/jcm14155562

**Published:** 2025-08-07

**Authors:** Mathias Chea, Chloé Bourguignon, Antonia Perez-Martin, Jean-Christophe Gris

**Affiliations:** 1 Department of Hematology, CHU Nîmes, Montpellier University, 30029 Nîmes, France; mathias.chea@chu-nimes.fr (M.C.); chloe.bourguignon@chu-nimes.fr (C.B.); 2 Faculty of Pharmaceutical and Biological Sciences, Montpellier University, 34090 Montpellier, France; 3 UMR 1318 INSERM, INRIA, Université de Montpellier IDESP, Montpellier University, 34090 Montpellier, France; antonia.perez.martin@chu-nimes.fr; 4 Department of Vascular Medicine, University Hospital, 30029 Nîmes, France; 5 Department of Obstetrics, Gynecology and Perinatal Medicine, I.M. Sechenov First Moscow State Medical University, 119991 Moscow, Russia

“There is a tendency among young men about hospitals to study the cases, not the patients and, in the interest they take in the disease, lose sight of the individual.” (Sir William Osler, 1885) [1].

Venous thromboembolism (VTE) is a chronic illness which includes both deep vein thrombosis (DVT) and pulmonary embolism (PE). A growing body of evidence suggests complex causal activating processes between the coagulation and immune systems, with innate immune cells contributing to thrombus formation (immunothrombosis) [2]. Activated leukocytes can generate procoagulant circulating tissue factor-positive microvesicles which participate in thrombus formation and growth [3]. VTE is a multicausal disease believed to be triggered by interactions between several factors which may be cumulative or synergistic, strong or weak, persistent or transient, with a significant proportion of unprovoked events [4]. Just how the inherited and acquired particularities of the coagulation system, prone to provoking an increase in thrombin (the coagulation terminal enzyme) generally known as “thrombophilia,” contribute to the risk of VTE is not fully identified.

Emerging data, sometimes perceived as provocative, raise the question of whether VTE should be included, or at least partly included, in the general field of psychosomatic medicine. The term “psychosomatic medicine” also has attracts meanings and connotations, with varying degrees of popularity. It may be defined as “a comprehensive, interdisciplinary framework for assessing the psychological factors affecting individual vulnerability as well as the course and outcome of a disease, biopsychosocial considerations of patient care in clinical practice, and specialist interventions to integrate psychological therapies into the prevention, treatment and rehabilitation of medical disease” [5]. There is a tendency to rely only on “hard data” in clinical medicine [5]. However, cumulated data on the significance of stress-induced hypercoagulability in clinical medicine, as reviewed by R. von Känel [6], is paving the way for this convergence, often perceived as heretical.

Many studies have described an association between psychosocial stress and arterial, atherothrombotic events [6]: numerous prospective cohort studies evidence an association between stress/hemostatic factors of a hypercoagulable state and an increased incident risk of and poor prognosis for atherosclerotic cardiovascular disease. While mendelian randomization studies suggesting that these associations are partly causal, data on VTE are more limited.

Epidemiological data focused on global VTE incidence are still limited.

Beginning with cohort studies, data from the Danish national registry found that post-traumatic stress disorder was associated with VTE, providing evidence that stress-related psychopathology is a risk factor for abnormal symptomatic venous clotting [7]. The Nurses’ Health Study II, analyzing new-onset VTE over 22 years in 49,296 women, showed that the exposure to the traumatic events commonly associated with psychological distress and post-traumatic stress disorder (PTSD) were significantly associated with a greater risk of developing VTE [8] and that, in fully adjusted models, women with the highest PTSD symptoms had an almost 2-fold greater risk of VTE than women not exposed to trauma. We may note that the results of that study were similar, albeit attenuated, when adjusting for VTE-relevant medications, medical conditions, and health behavior.

Moving to trials, a questionnaire on stress was completed by 6958 Swedish men participating in a cardiovascular intervention trial from 1970 to 1973. Men with persistent stress had a greater risk of pulmonary embolism and, compared with manual workers, intermediate or high-level male white-collar workers and professionals showed an inverse relationship between occupational class and pulmonary embolism [9].

Examining systematic reviews and meta-analyses, individual-level data analysis from eight European prospective cohort studies participating in the Individual–Participant Data Meta-analysis in Working Populations Consortium found that long working hours exceeding 55 h a week were associated with VTE, mainly DVT [10]. A systematic review in Iran concluded on a higher rate of deep vein thrombosis (DVT) in survivors of earthquakes compared to those of other disasters [11].

Among stress-generating circumstances is intimate partner violence (IPV). It affects at least 15% of women during their lifetime, resulting in highly significant stress, lifetime psychological violence being the most prevalent form of IPV. Although the lifetime risk of VTE is quite similar for men and women, women face a higher incidence during their reproductive years, mainly due to transient reproductive factors such as pregnancy, puerperium, and hormonal exposure, the most common being combined oral contraceptives (COCs) [4,12]. The recently developed, debatable concept of stress-induced coagulopathy [6] provides a mechanism suspected of triggering VTE not yet considered, which might participate in the genesis of many VTE events. We thus performed a multicenter, international, case–control study matched on age, country/region of residence, duration of COC intake, and their type to examine the relationship between the first VTE associated with COC intake and IPV (WAST-VTE study, 997 case–control pairs) [13]. IPV was evaluated using the WAST self-administrated questionnaire, and was more common among VTE cases than in controls (10.9% vs. 3.3%). After multivariate analysis, the adjusted conditional odds ratio (OR) was 3.72 (2.44–5.68). A sensitivity analysis using increasing WAST score thresholds confirmed the association. We then studied associations between the answers to the WAST items indicating verbal/psychological violence: after adjusting for all covariates, verbal and/or psychological violence were positive risk factors for VTE. This precisely focused study adds important data to the association of stress-generating conditions and VTE.

Epidemiological data focused on the recurrence of VTE incidence are also limited.

We performed one of the first multicenter, international, retrospective observational studies to examine the relationship between VTE recurrence and psychosocial factors (the VTE-WEAK study) [14]. We included 7754 women aged 18 to 55 with a history of VTE triggered by weak transient risk factors, such as COCs, pregnancy, puerperium, minor trauma, brief surgery, infection, and immobility. Among the cohort, 4772 women reported a suspected recurrence, with 1316 diagnoses confirmed. Stress and IPV were assessed using standardized questionnaires (PSS-10 and WAST) at the time of being evaluated for recurrence. Moderate and high levels of perceived stress and IPV were identified as independent predictors of recurrence. High perceived stress showed a particularly strong association (adjusted Odds Ratio (aOR): 10.03), whereas IPV also significantly increased the risk of recurrence (aOR: 1.95). These findings suggest a dose–response relationship and highlight the importance of addressing stress management in high-risk patients. The VTE-WEAK study thus provides valuable insights into the complex interaction between psychosocial and clinical factors in VTE recurrence, broadening our understanding of this multifactorial condition.

A prospective observational study in Switzerland investigated 271 consecutive patients referred for thrombophilia investigation with an objectively diagnosed episode of VTE. They had all completed the depression subscale of the Hospital Anxiety and Depression Scale (HADS-D) and, compared to patients with lower levels of depressive symptoms, those with high levels had a four-times-greater risk of recurrent VTE [15].

The findings highlight the critical need to incorporate stress and IPV screening into VTE risk assessments, addressing an often-overlooked aspect of patient care. Screening is based on tools, mainly questionnaires, that can be self-administered or administered by a trained examiner. Each of them and each administration procedure has its own limitations.

The Perceived Stress Scale (PSS), 10-item version: PSS-10 is a self-reported questionnaire designed to measure the degree to which situations in one’s life are appraised as stressful. It has been widely validated in various populations and is currently the most recommended, being practical and having the most satisfactory psychometric qualities. The 10-item version is the most widely used, being more reliable than the 4-item version (PSS-4). However, it only applies to the last month and does not consider previous striking events that could contribute to stress: another tool, the Stressful Life Events Screening Questionnaire (SLESQ), is a 13-item self-report measuring assesses lifetime exposure to traumatic events. These tools are highly subjective: besides self-report measures, stress response can also be measured with behavioral coding or via physiological measurements. It is recommended the latter be systematically included in studies to assess the individual consequences of the stress experienced, using hormones, cytokines, and white blood count panels.

The Woman Abuse Screening Tool (WAST) questionnaire is an 8-item, self-administered tool developed in Canada for screening domestic violence against women, which has been widely validated in English and French, among other languages. It is simple to use and is well received by patients, demonstrating very good acceptability. It only applies to the last 12 months and, again, does not consider past situations that may have contributed to chronic individual stress over a long period of time. Here too, combining this with the quantification of biological stress markers would enable a more objective assessment of the individual impact of the violent situations identified.

These data seem to reinforce the necessity for a holistic approach to VTE management, combining psychosocial evaluation with targeted interventions alongside traditional clinical strategies. Prospective studies are now warranted to identify and test relevant biomarkers and deepen our understanding of stress-induced coagulopathy, whose involvement in various clinical situations, characterized by a risk of VTE, needs to be better understood.

More globally, the association between psychological stress and VTE is currently not yet unraveled and seems to exist in both directions. For instance, association studies show that diagnosed depression seems to predict the risk of further VTE [16,17,18], and patients with a previous VTE event are exposed to a greater risk of depression [19,20,21].

Even studies performed on huge cohorts and after full adjustment on covariates, when available, only highlight associations with low levels of evidence and not the causes. One nationwide study in Sweden examined family associations between VTE and psychiatric disorders, using a pedigree-based approach examining the risk of psychiatric disorders in offspring from extended pedigrees according to the densities of VTE in pedigrees (482,184 analyzed pedigrees) [22]. The family study showed that several psychiatric diseases aggregate in the same families as VTE. Higher density rates of VTE, especially among females in pedigrees, thus demonstrated a statistically significant yet weak association in the offspring, with a higher risk of psychiatric disorders. Moreover, the rate of VTE in pedigrees was also significantly associated with depression and anxiety disorders, substance abuse, neurotic stress-related and somatoform disorders, behavioral syndromes associated with psychological disturbances and physical factors, adult personality disorders, and behavioral disorders in the offspring. By contrast, no association between familial rates of VTE and schizophrenia, organic mental disorders (including dementia), or bipolar disorders was observed. VTE thus shares familial susceptibility, albeit slight, with several psychiatric disorders.

Whilst maintaining an epidemiological approach, Mendelian randomization analyses measured variations in genes to examine the causal effect of exposure on an outcome. This design reduces both reverse causation and confounding, which often substantially impede or mislead the interpretation of the results of epidemiological studies. Using that technique, a huge collaborative study aimed to determine whether a genetic predisposition to major depressive disorder, bipolar disorder, or schizophrenia was associated with a greater risk of VTE [23]. Genetic correlations, using statistics from the largest genome-wide genetic meta-analyses for these diseases (the Psychiatric Genetics Consortium) and a recent genome-wide genetic meta-analysis of VTE (the INVENT Consortium) demonstrated a positive association between VTE and major depressive disorder. The same summary statistics were used to construct polygenic risk scores for the three psychiatric diseases in UK Biobank participants of self-reported white British ancestry. These were assessed for the impact on self-reported VTE risk using logistics regression, in sex-specific and sex-combined analyses. They highlighted significant positive associations between the polygenic risk of major depressive disorder and the risk of VTE in men, women, and sex-combined analyses, independent of known risk factors. Secondary analyses demonstrated that this association was not driven by those with lifetime experience of mental illness. Meta-analyses of individual data from six additional independent cohorts replicated the sex-combined association. The study [23] provides evidence for shared biological mechanisms leading to major depressive disorder and VTE. Among genes defining genetic predisposition to major depressive disorders found to be associated with the risk of VTE are genes involved in cytokine and immune response and those known to bind to the retinoid X receptor.

We currently have no available results from behavioral intervention trials testing the impact of clinical situations associated with a greater risk of VTE, or the risk of VTE recurrence.

The mechanism suspected of underlying venous thrombotic risk due to stress and mood disturbances is the induction of modifications in the biology of the vessel and the blood–vessel interface leading to a prothrombotic phenotype. This can be summarized as “stress-induced hypercoagulability” and has been extensively reviewed by R. von Kânel thanks to his strong personal inputs [6]. Putative peripheral actors and pathways include bone marrow thrombopoiesis, endothelial cells, circulating leukocytes and platelets, coagulation and fibrinolysis pathways, the complement system, and inflammatory mediators. Neuroendocrine pathways are the messengers linking the cerebral physiology modified by stress and the venous hemovascular pathophysiology. When the sympathetic nervous system is activated, it increases secretion of the transmitters epinephrine and norepinephrine. This activation of the hypothalamic–pituitary–adrenal axis increases cortisol and vasopressin secretion and parasympathetic vagal withdrawal. All these transmitters have significant effects on peripheral actors of the venous hemovascular biology and can tip the antithrombotic/prothrombotic balance in the prothrombotic direction.

Psychological stress thus progressively emerges as a new risk factor for VTE. Stress is a fundamental adaptive response which invokes the amygdala and the hypothalamus–pituitary–adrenal axis along with other brain regions. This state initially protected individuals by making them able to better react in the face of danger, but the individual constitutive and acquired characteristics that make it a risk factor for disease deserve to be understood. Henri Laborit, a founder of modern neuropsychopharmacology, developed the concept of inhibition of action [24,25], putting forward a theory regarding the necessity to counteract the negative consequences of defense mechanisms during behavioral inhibition. Laborit suggested that stress becomes pathogenic when individuals cannot initiate the instinctive, ancestral physical action associated with immediate survival, triggered by stress. The concept of allostasis emerged in the 1980s, under the impetus of two neuroscientists, Peter Sterling and Joseph Eyer [26]. This notion refers to all biological processes (metabolic, neuroendocrine, and vascular, among others) which enable the organism to return to a state of equilibrium following exposure to an exogenous factor. Allostatic load is a persistent deregulation or stimulation of this allostasis, with deleterious consequences for the organism (particularly within the cardiovascular system) leading to overall biological wear and tear [27,28,29]. However, despite widespread exposure to trauma, the incidence of post-traumatic stress disorder is relatively low, only one tenth, suggesting that either individual susceptibility or adaptability driven by epigenetic and genetic mechanisms is probably at play [30,31,32]. Prospective studies to analyze the role of perceived individual stress in venous thrombotic risk are now required. The individual characteristics modulating the effect of stress on venous thrombotic risk remain to be understood, and confirmation would lead to testing targeting therapeutic interventions. We feel that it is already necessary to make doctors and healthcare teams treating VTE aware of the psychological stress that may be present in patients, whatever form this stress may take, and include it in concerns as part of the etiological assessment.

## Abbreviations

VTE	venous thromboembolism
DVT	deep vein thrombosis
PE	pulmonary embolism
PTSD	post-traumatic stress disorder
IPV	intimate partner violence
COC	combined oral contraceptive
WAST	women abuse screening tool
HADS-D	hospital anxiety and depression scale
OR	odds ratio
aOR	adjusted odds ratio
UK	United Kingdom
